# Predictive approach of health indicators from the physical activity habits of active youth

**DOI:** 10.1038/s41598-024-62697-6

**Published:** 2024-06-06

**Authors:** Laura Moreno-Gonzalez, Samuel Manzano-Carrasco, Jose Luis Felipe, Antonio Alonso-Callejo, Leonor Gallardo, Jorge Garcia-Unanue

**Affiliations:** 1 IGOID Research Group, Faculty of Sport Sciences, University of Castilla-La Mancha, Toledo, Spain; 2 Department of Communication and Education, Universidad Loyola Andalucia, Sevilla, Spain

**Keywords:** Body composition, Physical fitness, Sport, Children, Adolescents, Health care, Health occupations

## Abstract

The aim of this study is to analyse the relationship between sport modalities practiced, physical fitness, body composition, and healthy habits in an active young population, using a statistical model for prediction. A total of 2255 (1528 boys and 727 girls) children and adolescents aged 6–17 years old who were involved in extracurricular sports from rural areas of Spain participated. Physical fitness was assessed through validated field test and, body composition was determinated using bioelectrical impedance analysis. Adherence to the Mediterranean diet was assessed by KIDMED questionnaire. The general sport variable was significant in VO_2_max when comparing the invasion and combat modalities to the reference level (court/net). The sex and age variables revealed significant differences in all physical fitness and body composition parameters. Health parameters, such as hours of additional practice, adherence to the Mediterranean diet, and previous experience, showed significant differences. The study concludes that the sport modality variables of training, sex, age, and maturational period have an impact on body composition and fitness parameters in this population. Therefore, by focusing on factors associated with lower values in health indicators, we can prevent health problems during adulthood, such as cardiorespiratory deficits.

## Introduction

Regular physical activity (PA) is one of the best strategies to promote health and well-being^1^. In this sense, PA plays a crucial role in the prevention and management of childhood illnesses^2^. Regular PA leads to improved outcomes in body composition, components of metabolic syndrome, cardiovascular fitness, and mental well-being^1^. Childhood and adolescence are crucial stages for acquiring and establishing healthy lifestyle habits, such as the regular practice of PA and the adoption of appropriate dietary patterns^3^, which are determinants of health from childhood to adulthood^4^.

The World Health Organisation (WHO) and the European Commission have established the promotion of PA as one of the priority objectives in society, especially in children and young people^5^. According to current guidelines, it is recommended that children and young people aged 5–17 years perform at least 60 min of moderate to vigorous physical activity (MVPA) per day^6^. Despite the evidence that PA has benefits, it is estimated that 81% of young Europeans do not adhere to this general recommendation^7^. Therefore, it is necessary to increase sports participation and to disseminate knowledge about the positive effects of sports on health^8^ from public health organizations^9^.

The WHO states that participation in PA varies by region and geographic location^7^. Participation in sports programmes depends to a large extent on regular opportune situations for the development of these activities and a support network, such as family, school, and community^10,11^. In summary, when an afterschool sports programmes are developed, influential factors, good practices, the interests of the population, and principles that help to improve the quality of these programmes should be taken into account^12^.

The literature shows that body composition, physical fitness, and nutritional habits are determining factors for health parameters in children and young people^13^. Hence, it is important to know that the sport discipline practiced can lead to different morphological, physical, and physiological adaptations^13,14^. Moreover, the variety in the frequency and intensity of exercise causes a specific adaptive response^15^. Children with more average hours in a week to MVPA show higher values of physical fitness and anthropometric parameters^16,17^. However, due to the limited literature we have found on the subject, the impact of these health indicators has yet to be determined. Thus, the aim of this study is to analyse the relationship between sport modalities practiced, physical fitness, body composition, and healthy habits in an active young population, using a statistical model for prediction.

## Methods

### Study design and participants

This cross-sectional study focused on children and adolescents utilised data collected from May 2018 to March 2023 for the Active Health project^3,18^. A convenience sample of 2255 children and adolescents aged 6–17 years old participated in this study. The sample was divided based on sex (1528 boys and 727 girls), age range (6–9, 10–13, 14–17 years), and sports modality (court or net, field or striking/fielding, individual and invasion/territorial games), according to the classification established in other studies^19^. Table 1 presents descriptive data on the physical fitness and body composition of the sample, categorized by sex, age group, and general sport classification.

**Table 1:** Descriptive values of the dependent variables for sex, age group and general sport classification.

			VO_2_max (ml/kg/min)				Fat mass (%)				Muscle mass (%)				Handgrip strength (kg)				Vertical jump (cm)				MFR (kg/kg)				HBMI (kg/kg/m^2^)			
			X̅	SD	Me	IQR	X̅	SD	Me	IQR	X̅	SD	Me	IQR	X̅	SD	Me	IQR	X̅	SD	Me	IQR	X̅	SD	Me	IQR	X̅	SD	Me	IQR
Boys	Court/net	6–9	47.2	4.2	47.6	6.3	26.0	8.1	24.6	9.8	69.8	7.6	70.8	9.1	14.3	3.9	14.6	5.3	17.5	4.3	17.5	5.0	1.1	0.4	1.0	0.6	0.7	0.2	0.7	0.3
	Court/net	10–13	45.9	4.8	46.4	6.5	23.2	8.5	20.0	13.1	72.7	8.0	75.6	12.3	21.2	5.6	20.6	8.1	23.6	5.5	24.1	6.9	1.5	0.6	1.5	1.0	1.1	0.3	1.1	0.4
	Court/net	14–17	47.6	4.2	47.5	6.9	19.5	5.8	17.9	6.3	76.4	5.5	77.9	6.0	31.4	10.2	34.0	11.7	30.3	7.0	30.0	9.8	1.9	0.6	1.9	0.8	1.5	0.5	1.7	0.6
	Invasion	6–9	49.4	3.7	49.8	4.8	22.2	6.0	21.3	6.8	73.4	5.6	74.3	6.2	12.9	3.3	12.8	4.7	19.5	4.1	19.5	6.0	1.4	0.9	1.3	0.5	0.7	0.2	0.7	0.2
	Invasion	10–13	47.9	5.1	48.1	7.7	23.5	7.2	21.7	9.8	72.4	6.8	74.1	9.2	22.3	6.2	21.1	7.1	23.9	5.7	23.1	7.3	1.5	0.5	1.4	0.8	1.1	0.3	1.1	0.4
	Invasion	14–17	47.7	6.1	47.6	9.7	19.6	6.2	17.7	7.5	76.3	5.9	78.1	7.0	35.0	8.8	34.3	11.5	32.6	6.8	32.4	8.5	1.9	0.7	1.9	0.9	1.6	0.4	1.6	0.5
	Individual	6–9	48.8	4.5	49.4	6.2	23.7	5.6	21.8	6.3	72.0	5.3	73.7	5.7	14.1	4.0	13.1	4.0	19.5	3.7	19.7	3.4	1.2	0.3	1.3	0.5	0.8	0.2	0.8	0.2
	Individual	10–13	46.5	5.5	46.4	5.7	23.5	8.5	20.9	8.4	72.4	8.0	74.9	7.9	21.1	5.6	20.9	6.5	23.6	5.8	23.2	7.4	1.5	0.6	1.5	0.7	1.1	0.3	1.1	0.3
	Individual	14–17	52.0	6.5	51.7	9.7	18.3	4.4	17.6	3.4	77.5	4.2	78.1	3.2	35.6	8.8	38.4	9.7	34.0	5.0	34.2	6.0	2.0	0.6	1.9	0.5	1.7	0.5	1.7	0.7
	Combat	6–9	48.5	3.7	49.1	6.2	23.5	5.7	21.9	8.2	72.1	5.4	73.6	8.0	12.3	3.2	11.6	4.6	17.1	4.6	16.5	5.1	1.2	0.3	1.2	0.6	0.7	0.2	0.7	0.2
	Combat	10–13	48.0	6.3	50.3	7.5	22.2	5.1	20.6	5.3	73.7	4.9	75.1	5.1	20.2	7.9	18.5	11.3	27.5	8.4	29.8	12.7	1.5	0.4	1.5	0.5	1.0	0.4	1.0	0.4
	Combat	14–17	44.3	7.5	43.4	10.5	18.8	6.1	18.2	3.8	77.0	5.9	77.7	3.6	37.3	11.6	40.9	13.0	36.0	8.9	38.4	7.4	2.0	0.7	1.9	0.5	1.7	0.5	1.9	0.7
Girls	Court/net	6–9	44.8	3.4	44.9	4.3	27.1	6.0	26.3	6.2	69.1	5.7	69.8	5.8	12.4	3.1	13.1	4.9	17.9	4.6	18.3	7.9	1.0	0.3	1.0	0.3	0.7	0.2	0.7	0.2
	Court/net	10–13	43.6	4.6	43.0	6.7	27.8	5.9	26.7	8.2	68.5	5.5	69.6	7.8	22.3	5.0	22.1	6.6	22.8	4.9	22.7	7.1	1.1	0.3	1.0	0.4	1.1	0.2	1.1	0.4
	Court/net	14–17	40.4	4.0	40.6	4.8	28.4	5.3	27.3	7.1	68.0	5.0	68.9	6.7	24.1	3.8	23.7	4.2	25.3	5.5	24.4	6.8	1.0	0.2	1.1	0.3	1.1	0.2	1.1	0.3
	Invasion	6–9	47.5	4.3	47.6	4.9	25.2	5.6	24.2	7.6	70.8	5.3	71.9	7.1	13.0	2.9	13.7	4.2	19.2	5.4	19.6	7.0	1.2	0.5	1.1	0.6	0.8	0.2	0.8	0.3
	Invasion	10–13	45.4	4.3	45.5	4.9	26.4	5.1	26.4	6.6	69.8	4.9	69.9	6.2	19.8	5.2	19.8	8.6	22.3	5.4	22.9	6.4	1.1	0.3	1.1	0.4	1.0	0.2	1.0	0.3
	Invasion	14–17	38.8	4.7	38.7	4.9	28.5	6.5	28.1	5.3	67.8	6.2	68.2	5.0	24.2	6.1	25.0	5.3	26.1	5.7	24.3	8.4	1.1	0.4	1.0	0.3	1.1	0.2	1.1	0.2
	Individual	6–9	46.9	3.5	47.2	4.2	25.3	5.3	24.6	7.2	70.8	5.0	71.4	6.8	12.2	3.2	12.1	4.2	17.5	4.0	17.2	5.6	1.1	0.5	1.1	0.4	0.7	0.2	0.7	0.2
	Individual	10–13	44.2	4.0	44.0	5.2	26.4	5.7	25.2	8.5	69.8	5.4	70.9	8.0	19.5	5.0	19.0	7.1	22.3	4.6	22.7	5.7	1.1	0.3	1.1	0.5	1.0	0.3	1.0	0.3
	Individual	14–17	41.2	5.4	40.6	6.5	28.2	7.0	26.7	9.1	68.2	6.7	69.6	8.7	25.6	4.3	25.9	4.7	24.7	4.2	25.2	4.6	1.1	0.3	1.0	0.5	1.2	0.2	1.2	0.3
	Combat	6–9	45.0	4.2	44.4	6.8	24.0	6.4	23.4	10.3	71.9	6.1	72.5	9.8	11.2	3.1	9.8	4.2	18.2	4.3	18.4	5.8	1.3	0.6	1.2	0.5	0.6	0.1	0.6	0.2
	Combat	10–13	41.7	2.7	41.0	3.5	27.0	8.9	25.3	12.8	69.2	8.4	70.9	12.0	19.8	5.5	18.8	7.1	22.4	5.6	23.3	9.4	1.2	0.6	1.1	0.6	1.0	0.3	0.9	0.3
	Combat	14–17	30.5	10.3	26.7	9.8	30.1	10.6	28.8	10.6	66.3	10.0	67.6	9.9	24.0	10.0	26.8	9.8	19.8	0.4	19.7	0.4	1.0	0.5	1.0	0.5	1.0	0.3	1.0	0.3

*X̅* mean, *SD* standard deviation, *Me* median, n; *IQR* interquartile range, *VO*_*2*_*max* maximal oxygen uptake, *MFR* muscle-fat-ratio, *HBMI* handgrip strength to body mass index; 6–9, 10–13, 14–17: age group classification; court/net, invasion, individual, combat: general sport classification.

All participants who were part of the study were enrolled in one of the municipal sports schools in a central and rural region of Spain. They engaged in an extracurricular sport activity, in its different modalities, at least two days a week for a minimum of 1 h each day. Among these sports modalities and following the classification specified by Ellis^19^, 1298 participants played invasion sports (football, basketball, futsal), 298 participants played court or net sports (tennis, paddle tennis, badminton), and 65 participants played fight/combat sports (judo, karate…). In addition, taking into account a recent study^20^, individual or travel sports such as athletics, cycling, motorcycling, or swimming were added to this classification, in which 594 participants took part. All participants enrolled in sports schools were invited to participate in this project, but only those who successfully completed all the tests were considered for analysis.

The study was conducted according to the standards of the Declaration of Helsinki (World Medical Association, 2013) and the European Community Guidelines for Good Clinical Practice (111/3976/88 July 1990). The instructions for clinical research in humans governed by the Spanish legal framework were followed (Royal Decree 561/1993). This study was accepted by the Ethics Committee for Clinical Research of the University Hospital of Toledo (Spain), which approved the project (Ref.: 1038/28062023).

### Procedures and measurements

Once the study protocol had been approved, each participant underwent an individual assessment before the training session for the sport modality they were enrolled in. The test lasted between 60 and 90 min and was conducted in groups of 12–14 participants. Body composition and physical fitness tests were administered by qualified personnel and according to the protocol established in the Active Health project. In addition, the physical fitness tests were administered in such a way that fatigue did not influence the performance of the subsequent assessments. Participants and their parents or legal guardians were informed of the research objectives and test characteristics before the study began. They were required to give their informed consent prior to their son’s and/or daughter’s participation in the testing.

### Physical fitness

To assess the different parameters of physical fitness, an adapted version of the extended Assessing Levels of Physical Activity (ALPHA) health-related fitness battery for children and adolescents^21^ was used. The ALPHA-Fitness battery, established by Ruiz et al.^21^, is structured around three core components: aerobic capacity, musculoskeletal strength, and body composition. Researchers conducted all the fitness tests, and they were performed in the following sequence:

To evaluate upper-body muscular strength, a handgrip strength test with hand dynamometer with adjustable grip (Constant R Model: 14192-709E, China) was employed. Participants were instructed to maintain a full-extension elbow position and exert a continuous maximum force with their hands for a duration of 3 s. The test was performed alternately with the dominant and non-dominant hand, and participants were allowed a 30-s rest period between attempts. The score for each individual's best performance with his or her dominant hand was recorded to the nearest 1 g and expressed in kg as an absolute value^21^. The vertical jump test was used to determine the strength of the lower body muscles. During a countermovement jump (CMJ) with arm swing, the maximum vertical jump height was measured with an accuracy of 0.1 cm using photoelectric cells formed by two parallel bars (Optojump, Microgate, Bolzano, Italy). The CMJ arm swing test is a reliable and valid field test for determining muscular fitness^22^. This system measures the time of flight as the interval between take-off and landing. Prior to data collection, the CMJ test was explained to each participant. Participants were directed to execute jumps with maximal vertical height, involving a swift downward preparatory eccentric movement, allowing freedom of arm movement. Each participant successfully executed three jumps, and the highest jump among them was selected as the ultimate performance measurement.

Cardiorespiratory fitness was assessed by performing a maximum incremental field test (20-m Shuttle-Run Test). Participants were instructed to run back and forth between two lines spaced 20 m apart while maintaining a pace emitted by acoustic signals from a portable speakerphone. The test began with an initial speed of 8.5 km h^−1^ and was gradually increased by 0.5 km h^−1^ each min, according to the protocol established by Leger et al.^23^. The test was considered complete when the participant failed to reach the end of the lines in sync with the audio signals on two consecutive occasions. Alternatively, the test concluded when the participant stopped due to fatigue. The results were transformed in stages of 1-min duration, and the maximal oxygen uptake (VO_2_max) was estimated using the formula by Leger et al.^23^:

$${VO}_{2}max\left(\text{ml}\cdot {\text{kg}}^{-1}\cdot {\text{min}}^{-1}\right)=31.025+3.248\cdot {X}_{1}-3.248\cdot {X}_{2}+0.1536\cdot {X}_{1}\cdot {X}_{2}$$

where the final speed is X1 (km h^−1^) and age is X2 (year as the lower rounded integer). The test was carried out just once, and it was done at the end to ensure that the participants' performance and fatigue would not affect the results.

### Body composition

Body composition was indirectly measured using a portable segmental analyser of multifrequency bioimpedance analysis (BIA) (TANITA MC-780, Tanita Corp., Tokyo, Japan), which has been clinically verified to be accurate and reliable and to provide highly reproducible results^24^. Weight (kg), fat mass (kg and %), and muscle mass (kg and %) data obtained by BIA were used. Height (cm) was assessed with a height rod (Seca 214, Hamburg, Germany). Body mass index (BMI) was calculated with the weight (kg) divided by squared height (m). Moreover, the appendicular skeletal muscle mass (ASMM) was calculated by the sum of the muscle mass of four limbs. According to Salton, et al.^25^, the muscle-to-fat ratio (MFR) (MFR = ASMM [kg]/fat mass [kg]) was calculated. Assessments were carried out with clothes on and barefoot. Finally, the handgrip strength-to-BMI (HBMI) ratio was estimated with the handgrip strength (kg) and BMI (kg/m^2^).

### Adherence to the Mediterranean diet

Adherence to the Mediterranean diet (MD) was determined using the KIDMED questionnaire. This test was created and validated in the enKid study^26,27^. This instrument was created with a focus on evaluating adherence to the MD among youth and adolescents. It has been used in recent systematic reviews^28,29^, providing a rich contextual framework for interpreting the outcomes. KIDMED consists of 16 items: 12 items represent a positive score for the adherence to the MD, and the remaining 4 items represent a negative score. A positive answer to a question that involves greater adherence to the diet is worth + 1 point (Q1–Q5, Q7–Q11, Q13, and Q15). A positive answer to a question that means less adherence to the diet is worth − 1 point (Q6, Q12, Q14, and Q16). Negative answers do not score (a value of 0 is noted). The sum of all values from the administered test is considered as the KIDMED index and is categorised into three different levels: (1) low adherence (very low-quality diet, 0–3); (2) medium or moderate (improvement of the diet is needed, 4–7); and (3) high adherence (optimal adherence to the MD, 8–12)^27^.

### Statistical analysis

Data analysis was conducted in R version 4.2.2 (2022-10-31 ucrt) with RStudio 2022.12.0. Participants were classified into three groups based on their age (6–9, 10–13, and 14–17 years old). This classification was checked with a linear discriminant analysis (LDA). The LDA aims to represent a dependent variable through a linear combination of other variables, thereby enabling a more precise classification of cases into specific age groups. This process is crucial for evaluating the model's ability to accurately classify cases by age and its generalization to new data. This analysis and its practical implementations have been already studied^30,31^. The predictor variables included in the LDA model were those that should be further analysed as dependent variables. Data partition 80–20% was performed in order to divide the sample into train and test data, respectively. Data were centred and scaled before modelling the discriminant function. Table 2 illustrates the accuracy of the LDA with 82.4% of cases correctly classified on the test sample and 81% in the training sample. Thus, three groups were accepted as a division method for the age variable.

**Table 2:** LDA accuracy and division method for the age variable.

		TEST 20% n = 449				TRAIN 80% n = 1806			
		Predicted				Predicted			
	Age group	6	10	14		6	10	14	
Original (%)	6	87.3	12.7	0	n = 166	88.14	11.86	0	n = 666
	10	12	83.3	4.6	*n* = *216*	11.65	79.98	8.37	*n* = *824*
	14	1.5	14.9	83.6	*n* = *67*	0.32	34.18	65.51	*n* = *316*
		*n* = *172*	*n* = *211*	*n* = *66*	Cases correctly classified = 82.4%	*n* = *684*	*n* = *846*	*n* = *276*	Cases correctly classified = 81%

Marginal row results based on the percentage of success classification. Age group is refered to 6–9, 10–13, 14–17 years age group.

Data distribution was tested using the Kolmogorov–Smirnov test. The variables of weight, height, and BMI were non-normally distributed along the different groups. The choice of a Generalized Additive Model (GAM) for our analysis stems from its versatility in capturing nonlinear relationships among predictor variables and the response variable. Contrary to conventional linear models, GAMs incorporate smooth functions represented by B-splines with penalties, facilitating the modeling of complex nonlinear associations (Hastie & Tibshirani, 1990). This approach stands out as a robust tool for estimating such intricate relationships, particularly when the relationships resist simple predictor transformations or polynomial equations. Despite the interpretational challenges posed by the inclusion of smooth terms, GAMs offer significant advantages in flexibility and predictive performance compared to simpler linear models. Thus, the selection of GAMs for our analysis represents a well-considered choice based on their ability to effectively model nonlinear associations while acknowledging the broader landscape of available modeling techniques. The function gam(), from the package mgcv (version 1.8-41), was used for the model fit. The model was built with the predictor variables of general sport, skill sport, years of practice, KIDMED, sex, age, age groups, and the smoothed variables s(weight), s(height), and s(BMI). These variables were used to run the model with each dependent variable (VO_2_max, fat mass, muscle mass, handgrip strength, vertical jump, MFR, HBMI). The number of nodes used for the different models was k = 20 as number in which k-index was higher than 1 (p > 0.05). The method for estimating the smoothed parameter was the restricted maximum likelihood. Age groups (6,10, and 14) were included in the smoothed terms as a factor to allow the interaction between them. The final model for each dependent variable was as follows:

$$y={V}_{1}+{V}_{2}+{V}_{3}+{V}_{4}+ {V}_{5}+{V}_{6}+{V}_{7}+{V}_{8}+s\left({V}_{9} by {V}_{5}, k=20\right)+s\left({V}_{10} by {V}_{5}, k=20\right)+s\left({V}_{11} by {V}_{5}, k=20\right)$$

V_1_ = general sport; V_2_ = skill sport; V_3_ = sex; V_4_ = age; V_5_ = age groups; V_6_ = years of practice; V_7_ = KIDMED; V_8_ = hours per week; V_9_ = weight; V_10_ = height; V_11_ = BMI; s = smooth.

Accuracy and error of the model were tested by different methods: adjusted R2, standard deviation of the original variable, model standard deviation, deviance explained, mean absolute error, mean absolute percentage error, root mean square error, index of agreement, and Akaike information criterion.

## Results

Parametric coefficients of the GAM model are shown as estimate coefficient and standard error for each dependent variable according to the predictive variables and their levels (Table 3). Categorical variables with more than two levels are expressed as the estimate coefficient to the reference level. The reference levels for categorical levels are court/net (general sport), opened modality (skill sport), and 6–9 (age group).

**Table 3:** Parametric coefficients of the GAM model for dependent variables and approximate significance of smooth terms.

Parametric coefficients	VO_2_max		Handgrip_strength		Vertical_jump		Fat_mass		Muscle_mass		MFR		HBMI	
	Estimate	Std Error	Estimate	Std Error	Estimate	Std Error	Estimate	Std Error	Estimate	Std Error	Estimate	Std Error	Estimate	Std Error
(Intercept)	48.43**	1.05	7.47**	1.16	6.34**	1.35	35.40**	1.22	60.94**	1.15	0.84**	0.16	0.42**	0.06
general_sport (individual modality)	0.80	0.67	0.79	0.71	0.28	0.73	− 0.13	0.54	0.17	0.52	0.03	0.08	0.05	0.03
general_sport (invasion modality)	0.95**	0.28	− 0.06	0.30	0.09	0.31	− 0.24	0.23	0.24	0.22	0.05	0.03	− 0.01	0.01
general_sport (combat modality)	− 1.31*	0.55	0.17	0.58	0.50	0.60	− 0.41	0.45	0.37	0.43	0.09	0.07	0.00	0.03
skill_sport (closed modality)	− 0.20	0.66	− 0.50	0.70	− 0.40	0.72	0.19	0.54	− 0.17	0.51	0.01	0.08	− 0.04	0.03
Sex	− 2.76**	0.24	− 1.55**	0.26	− 1.59**	0.26	3.60**	0.20	− 3.25**	0.19	− 0.32**	0.03	− 0.08**	0.01
Age	− 0.58**	0.11	0.98**	0.11	1.35**	0.12	− 0.69**	0.09	0.66**	0.08	0.05**	0.01	0.05**	0.01
age_group (10–13)	2.42**	0.64	1.61**	0.57	1.31	0.89	− 4.26**	1.00	4.11**	0.94	0.05	0.12	0.07*	0.04
age_group (14–17)	0.85	1.27	0.17	1.19	1.11	1.83	− 3.20*	1.47	3.13*	1.40	0.19	0.16	− 0.04	0.09
hours_per_week	0.25**	0.04	− 0.01	0.04	0.16**	0.05	− 0.08*	0.03	0.08*	0.03	− 0.01	0.01	− 0.01	− 0.01
years_of_practice	0.26**	0.04	0.06	0.04	0.11**	0.04	− 0.04	0.03	0.04	0.03	0.01*	− 0.01	− 0.01	− 0.01
kidmed	0.10**	0.04	0.07	0.04	− 0.03	0.04	0.02	0.03	− 0.02	0.03	− 0.01	− 0.01	− 0.01*	− 0.01

Aproximate significance of smooth terms	F	edf	F	edf	F	edf	F	edf	F	edf	F	edf	F	edf
s(weight):age(6–9)	1.63**	0.97	1.55**	1.61	7.41**	1.00	39.48**	3.86	38.86**	3.80	6.18**	2.43	0.01	0.15
s(weight):age(10–13)	0.09	0.99	1.48**	1.58	0.58**	2.28	5.96**	4.95	5.92**	4.95	8.36**	0.99	0.54**	2.53
s(weight):age(14–17)	1.5**	3.86	1.00**	0.97	1.10**	3.07	0.26	1.94	0.28	2.01	0.11	0.92	0.82**	3.41
s(height):age(6–9)	2.01**	2.15	0.61**	0.88	4.45**	2.21	17.37**	4.70	17.49**	4.68	3.24**	2.39	4.34**	1.79
s(height):age(10–13)	1.94**	3.39	1.31**	3.38	3.05**	2.22	7.13**	0.99	7.36**	0.99	10.22**	2.25	1.96**	3.27
s(height):age(14–17)	1.00**	2.79	6.68**	5.88	0.89**	1.69	2.63**	2.98	2.74**	3.01	1.77**	2.74	9.00**	5.73
s(bmi):age(6–9)	0.47**	1.75	− 0.01	− 0.01	− 0.01	− 0.01	0.15	1.52	0.14	1.48	0.06	0.68	5.86**	0.99
s(bmi):age(10–13)	14.54**	3.80	1.49**	4.10	6.15**	3.77	3.86**	4.64	3.88**	4.68	1.61**	2.49	2.94**	2.54
s(bmi):age(14–17)	2.75**	5.77	1.04**	2.68	1.65**	6.00	18.37**	3.07	17.35**	3.14	8.32**	3.53	1.10**	3.23

*VO*_*2*_*max* maximal oxygen uptake, *MFR* Muscle-fat-ratio, *HBMI* handgrip strength to body mass index, *Estimate* regression coefficients, *Std Error* Standard Error, *F* statistic; *Significant differences (p < 0.05); **Significant differences (p < 0.01), *edf* Effective degrees of freedom. The reference intercept of the general_sport group corresponds to the court/net modality, while the intercept of the skill_sport corresponds to the opened modality. The intercept of the sex corresponds to the girls group, and the intercept of the age corresponds to the 6–9 group. Regarding the hour_per_week, years_of_practice, and kidmed groups, the intercept is set at 0.

**Figure 1:**
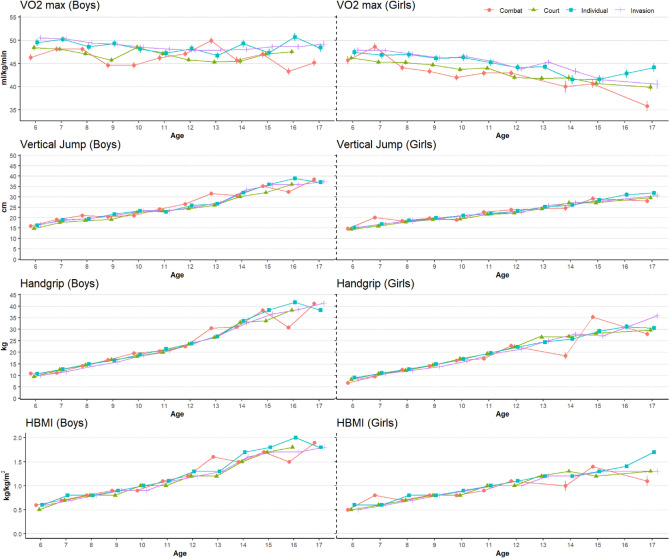
Predicted values and standard error of physical fitness variables for boys and girls. Predicted points are estimated based on the mean height and weight of each age and general sport classification. *VO*_*2*_*max* Maximal oxygen uptake, *HBMI* Handgrip strength-to-body mass index.

**Figure 2:**
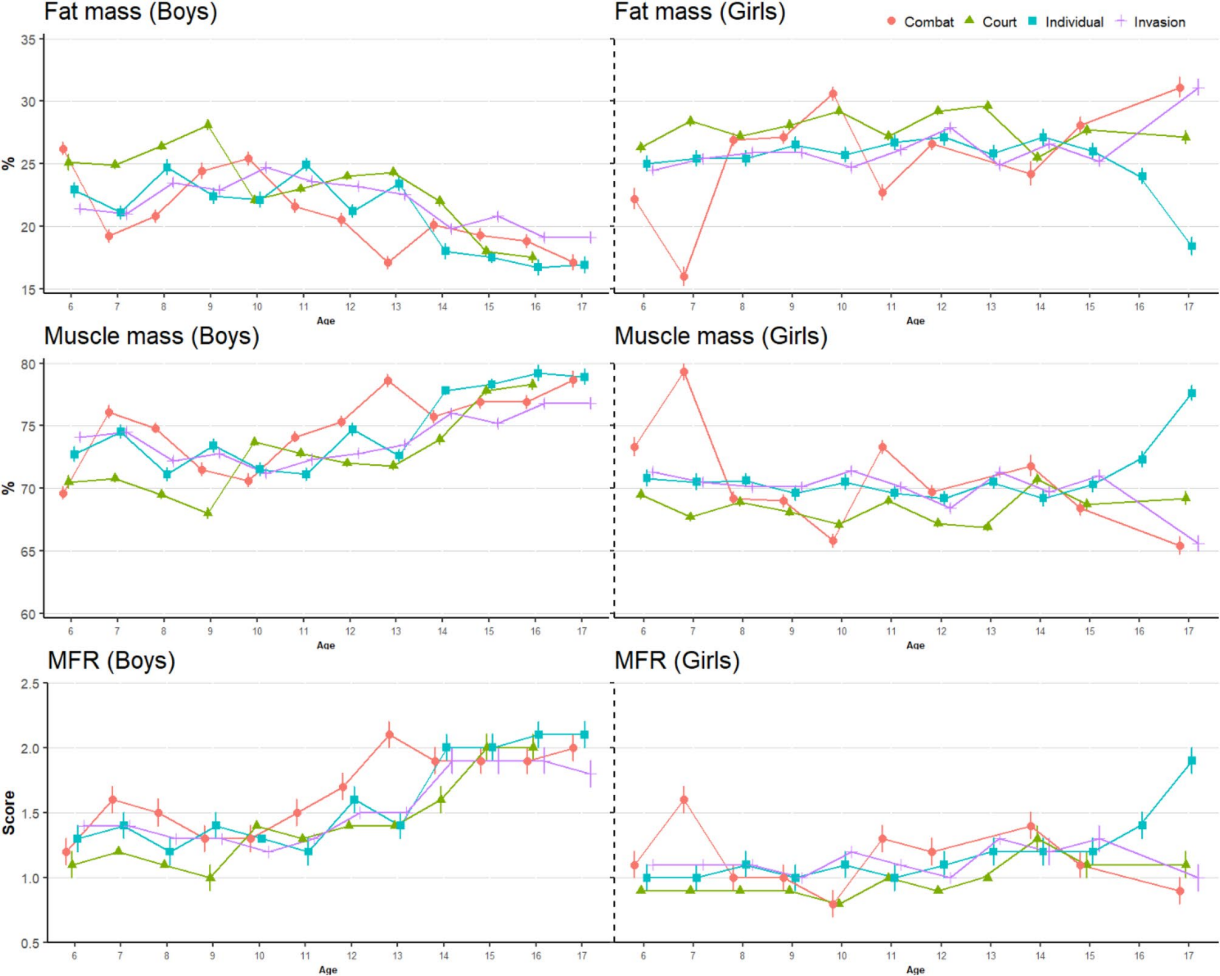
Predicted values and standard error of body composition variables for boys and girls. Predicted points are estimated based on the mean height and weight of each age and sport classification. *MFR* Muscle-fat-ratio.

Concerning VO_2_max (as shown in Table 3), significant differences were observed in the general sport variable, particularly when comparing invasion and combat modalities with the reference level (court/net wall). In addition, both sex and age variables demonstrated significant differences in all physical fitness and body composition parameters. The predictions of physical fitness and body composition based on mean values for each age, sex, and general sport classification are shown in Figs. 1 and 2, respectively. The results presented in Table 4 illustrate the performance metrics of various models across different physiological variables. R2 adj. values indicate the proportion of variance explained by the models, with values ranging from 0.42 for VO_2_max to 0.78 for fat mass, muscle mass, and handgrip strength. Standard deviations (SD) in the original and GAM-fitted data are noticeably reduced for most variables, suggesting improved model precision. Deviation percentages (Dev %) highlight the extent of improvement achieved by the GAM approach. Mean absolute error (MAE), mean absolute percentage error (MAPE), and root mean square error (RMSE) provide insights into the accuracy of predictions, showing generally low error rates across variables. The index of agreement (IOA) indicates strong agreement between observed and predicted values. Overall, the application of GAM models demonstrates promising results in enhancing the accuracy and precision of predictions for physiological variables, as evidenced by the reduction in errors and improved agreement with observed data.

**Table 4:** Model accuracy, error and standard deviation.

	R2 adj	Original SD	GAM SD	Dev (%)	MAE	MAPE	RMSE	IOA	AIC
VO_2_max	0.42	5.19	3.36	0.43	3.07	0.07	3.92	0.77	12,650.32
Handgrip_strength	0.78	9.04	8	0.79	2.9	0.15	4.17	0.94	12,917.68
Vertical_jump	0.6	6.85	5.28	0.6	3.35	0.16	4.32	0.86	13,080.14
Fat_mass	0.78	6.85	6.05	0.78	2.35	0.12	3.18	0.94	11,719.91
Muscle_mass	0.78	6.46	5.68	0.78	2.24	0.03	3.03	0.93	11,500.14
MFR	0.42	0.63	0.41	0.43	0.24	0.01	0.48	0.76	3157.65
HBMI	0.74	0.4	0.34	0.74	0.15	0.15	0.2	0.92	− 722.76

*R2 adj* Adjusted R2, *Original SD* Standard deviation of the original variable, *GAM SD* Model standard deviation, *Dev (%)* Deviance explained, *MAE* Mean Absolute Error, *MAPE* Mean Absolute Percentage Error, *RMSE* Root Mean Square Error, *IOA* Index of Agreement, *AIC* Akaike Information Criterion, *VO*_*2*_*max* maximal oxygen uptake, *MFR* muscle-fat-ratio, *HBMI* handgrip strength to body mass index.

## Discussion

The present study aimed to analyse the relationship between sport modalities practiced, physical fitness, body composition, and health habits in an active young population, using a statistical model for prediction. The main finding was that the sport modality significantly predicted VO_2_max. Additionally, variables such as sex, age group, hours per week, and years of sports practice were found to be the best predictors of physical fitness and body composition. For this reason, given that physical fitness is considered to be a useful marker of health in childhood and adolescence^32^, it is possible to estimate health-associated values through a practical battery of field tests in a young population.

### Physical fitness

Regular sports practice from an early age influences the peak of VO_2_max^14^. This influence is attributed to the varying intensities and physiological stimuli inherent in each sport modality^14^. Our predictive model (Table 3) indicates that invasion sports had higher VO_2_max values compared to court or net sports. The scientific literature has shown a positive association between invasion sports and musculoskeletal, cardiovascular, and metabolic adaptations, primarily due to their high aerobic component^33,34^. Consequently, the physiological adaptations resulting from the demands of a specific sport can explain these differences. However, our results revealed higher cardiorespiratory fitness values in court/net athletes compared to those practicing combat sports. This discrepancy can be associated with the explosive physiological patterns characteristic of combat sports, which do not emphasise training based on aerobic capacity^14^. Regarding individual sports, no significant results were observed in the aerobic capacity test. These findings can be attributed to various factors, including the potential influence of growth, development, and genetics, which are more determinant than the impact of the sport discipline^35^.

On the other hand, all fitness variables had a significant influence when analysed by sex, showing a decrease in girls compared to boys. These disparities primarily stem from the pivotal role of sexual maturation, as boys exhibit higher testosterone levels than girls during maturing ages^36^. In addition, these differences can strongly affect the development of physical and sporting skills^37^. Similarly, age was also a significant predictor of physical fitness values. Our results showed a decrease in VO_2_max (Fig. 1). This agrees with the study by Patel^37^, in which younger subjects showed a higher resting heart rate and a higher maximal heart rate, which is directly associated with higher VO_2_max values. Additionally, this decline in VO_2_max may be influenced by increased adiposity relative to body weight^17^. The opposite effect is observed in the muscle strength results. As athletes get older, their values in the handgrip strength and vertical jump tests increase. Biological maturity is a key factor in variances in physical fitness^38^.

Hence, these changes in muscle strength tests are related to the hormonal changes in puberty^38^. This gain is more pronounced in males, who experience an increase in testosterone levels of almost three times^36^. This disparity may explain why more mature participants demonstrate better results in strength tests, especially males.

Previous research has demonstrated that regular participation in sports and PA is associated with improved physical fitness^16^. The type of sport, intensity, frequency, and longer training sessions are associated with positive values for cardiorespiratory fitness and muscular strength^39^. In this sense, Table 3 shows that the frequency of sports practice and previous sports experience significantly increases maximal oxygen consumption and performance in the vertical jump test. The levels of VO_2_max and vertical jump height improve with each additional hour of practice per week, as well as with each accumulated year of sports experience. Finally, the model predicted higher values for VO_2_max and HBMI ratio when athletes had higher adherence to the MD. The literature review demonstrated the importance of adherence to the MD in fitness parameters^3,40^. This can be attributed to the improvement in body composition and cardiorespiratory profile resulting from the physical activity in which the participants engage^41^. Therefore, early adherence to the MD is crucial to prevent an increased risk of cardiovascular disease, obesity, or metabolic syndrome in adulthood^42^. In conclusion, the combination of high adherence to the MD and an active sport practice seems to provide the highest protection against cardiometabolic risk^43^.

### Body composition

Despite the research affirming that sport type influences and generates specific adaptations in anthropometric and physiological aspects in children and young people^14,44^, no significant differences were found in our results between sport type and body composition. This effect can be explained by not considering whether the subjects practiced different sports modalities, which disregarded the anthropometric adaptations produced by the practice of various sports. However, our results revealed differences in all body composition variables examined by sex. These findings are influenced by genetic and physiological distinctions between sexes^14^. After puberty, girls tend to increase in body fat mass, whereas boys show an increase in lean mass^45^, the latter associated with elevated testosterone levels^36^. This pattern would explain why boys have a greater ability to achieve higher levels of strength and cardiorespiratory fitness. Similar to the physical fitness parameters, age also behaves as a good predictor of muscle mass, fat mass, MFR, and HBMI. Athletes in the 10–13 and 14–17 years age groups showed higher values of muscle mass and lower values of body fat mass with respect to the 6–9 years group, especially in boys (Fig. 2). This trend in body composition may be attributed to their active participation in physical activities.

Significant differences were found between regular sports practice and body composition. With each additional hour of sports practice, there is a decrease in the percentages of fat mass and MFR, and an increase in muscle mass. Ara, et al.^16^ suggest that engaging in at least 3 h of sports per week in children is effective in reducing total and regional fat mass while increasing total lean mass. This research has certain limitations that need to be considered. The sample size in our study displays a notable sex imbalance, with significantly more boys (1528) than girls (727), potentially introducing bias into our findings. Furthermore, differences in sample size across various sports and age groups may have affected outcomes, considering factors like physical fitness, sport-specific physiological changes, anatomical characteristics, and the pubertal stage of each subject. Despite this imbalance, our sample size remains substantial and likely reflects societal norms regarding sports participation frequency, possibly justifying these disparities. It is crucial to note that our observational study design means sample selection is not controlled. This underscores the significance of acknowledging that secondary sports could lead to physiological and physical changes in individuals, emphasizing the necessity of incorporating these aspects into future research.

The results of the study conclude that the specific sport modality, frequency, intensity, and longer training affect body composition and fitness parameters in the child population. Our results reaffirm the existence of a relationship between regular participation in sports activities and anthropometric measurements, a powerful indicator of health. Therefore, this research provides further evidence for policy makers and researchers engaged in the promotion and development of active and healthy behaviours to take into account the importance of multilateral progress during the development of children. It is important to focus on sports and other factors that are associated with lower values in health indicators, such as cardiorespiratory fitness in combat sports. Strategies should be aimed at athletes who play these sports in order to achieve optimal specific values for children, taking into consideration factors such as sex, chronological age, and maturational period. In this way, possible health problems due to a cardiorespiratory-level deficit in adulthood could be avoided.

### Supplementary Information

Supplementary Information.

## Data Availability

All the data of the study can be found in the Supplementary File “SF1. Data of the Study”.
